# Supplementary material for: Gestational Diabetes Mellitus Treatment Schemes Modify Maternal Plasma Cholesterol Levels Dependent to Women´s Weight: Possible Impact on Feto-Placental Vascular Function
Source: Nutrients. 2020 Feb 17;12(2):506. doi: 10.3390/nu12020506 (PMC7071311; doi:10.3390/nu12020506)
Supplement: Supplementary file 1 [file nutrients-12-00506-s001.pdf]

nutrients

MDPI

Supplementary figures

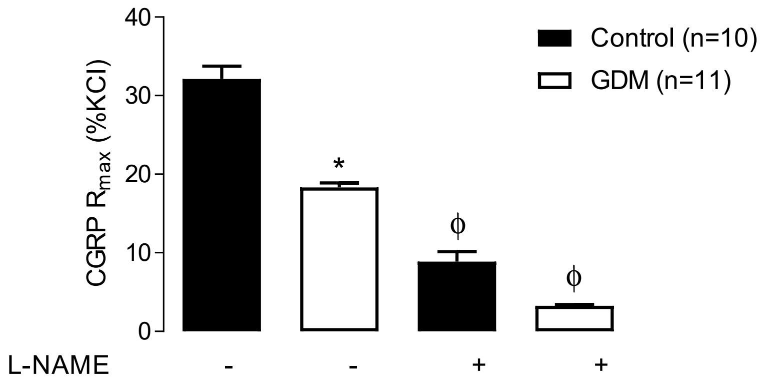

Figure S1. Maximal relaxation endothelium-dependent of human umbilical vein rings from nonobese control and with gestational diabetes mellitus women in response to CGRP.

Human umbilical vein rings were obtained at term from nonobese pregnant women from the control (C) group or those with gestational diabetes mellitus (GDM). Relaxation was determined in umbilical vein rings from the C (n=10) or GDM (n=11) groups in response to calcitonin gene-related peptide (CGRP, 0.001- 1000 nmol/L, 5 minutes). Rings were pre-constricted with 32.5 mmol/L KCl in the absence or presence of 100 μmol/L NG-nitro-L-arginine methyl ester (L-NAME, 20 minutes), and the percentage of inhibition of maximal dilation was graphed. Values are mean ± S.E.M. Significant differences were considered with $p<0.05$.* respect to C and φ respect to each group without L-NAME.

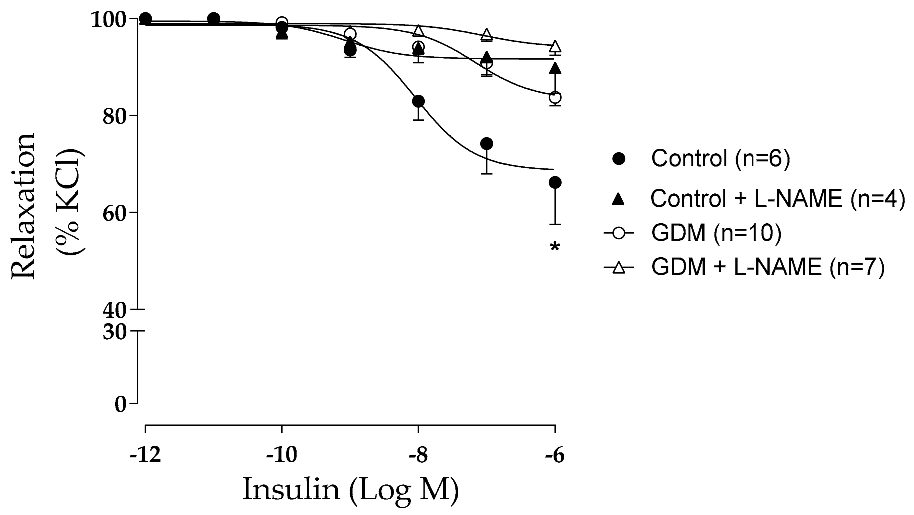

Figure S2. Dilation of human umbilical vein rings endothelial-dependent from nonobese control women and women with gestational diabetes mellitus in response to Insulin.

Human umbilical vein rings were obtained at term from nonobese pregnant women from the control (C) group or those with gestational diabetes mellitus (GDM). Relaxation was determined in umbilical vein rings from the C (n=6), C+ L-NAME (n=4), GDM (n=10) and GDM +L-NAME (n=7) groups in response to Insulin (0.001- 1000 nmol/L, 5 minutes). Rings were pre-constricted with 32.5 mmol/L KCl in the absence or presence of 100 μmol/L NG-nitro-L-arginine methyl ester (L-NAME, 20 minutes), and the percentage of inhibition of maximal dilation was graphed. Values are mean ± S.E.M. Significant differences were considered with $p<0.05$.* respect to C.

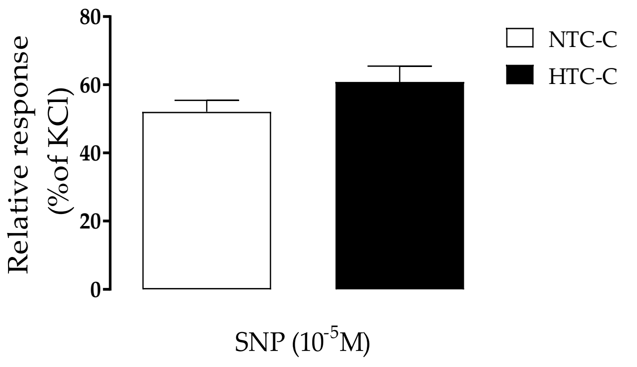

Figure S3. Dilation endothelial-independent of human umbilical vein rings from nonobese control women with normal and high cholesterol in response to Sodium nitroprusside (SNP).

Human umbilical vein rings were obtained at term from nonobese pregnant women from the control (C) group with normal (NTC) or high cholesterol levels (HTC). Relaxation was determined in umbilical vein rings from the NTC-C (n=11) and HTC-C (n=8) groups in response to SNP (10µmol/L, 5 minutes). Rings were pre-constricted with 32.5 mmol/L, and the percentage of inhibition of maximal dilation was graphed. Values are mean ± S.E.M.

© 2020 by the authors. Licensee MDPI, Basel, Switzerland. This article is an open access article distributed under the terms and conditions of the Creative Commons Attribution (CC BY) license (http://creativecommons.org/licenses/by/4.0/).
